# The Preoperative Prognostic Nutritional Index for the Prediction of Outcomes in Patients with Early-Stage Ovarian Clear Cell Carcinoma

**DOI:** 10.1038/s41598-020-64171-5

**Published:** 2020-04-28

**Authors:** Nobuhisa Yoshikawa, Kosuke Yoshida, Satoshi Tamauchi, Yoshiki Ikeda, Kimihiro Nishino, Kaoru Niimi, Shiro Suzuki, Fumitaka Kikkawa, Hiroaki Kajiyama

**Affiliations:** Department of Obstetrics and Gynecology, Nagoya University Graduate School of Medicine, Nagoya, Japan

**Keywords:** Ovarian cancer, Surgical oncology

## Abstract

The prognostic nutritional index (PNI), which reflects preoperative malnutrition, is useful for predicting the incidence of postoperative complications and has been reported in recent years to predict the long-term prognosis of various malignancies. The purpose of this study was to clarify the significance of PNI as a prognostic factor for early-stage clear cell ovarian carcinoma. A total of 82 patients with stage I–II (FIGO 2014) ovarian clear cell carcinoma undergoing primary surgery at our hospital from January 2005 to December 2017 were enrolled. PNI was calculated using the formula: 10 × serum albumin (g/ dL) + 0.005 × peripheral blood lymphocyte count (/mm^3^). Preoperative PNI exhibited relatively high area under the curve value (0.709) for 5 year survival, and the optimal cutoff value was 46.5. The overall survival was significantly shorter in the PNI-low group than in the PNI-high group. Multivariate analysis showed that high PNI was a significant independent prognostic factor for favorable prognosis (hazard ratio = 0.102, p = 0.010). There was no significant difference in recurrence-free survival between the two groups (p = 0.220), but the postrecurrence survival was significantly longer in the PNI-high group than in the PNI-low group (p = 0.0383). The preoperative PNI was a useful predictor of prognosis, even in early-stage ovarian clear cell carcinoma.

## Introduction

Ovarian clear cell carcinoma (OCCC) is one of the common histologic subtypes of epithelial ovarian cancer (EOC), accounting for approximately 20% of all EOC in Asian countries but only for 6% of all EOC in Western countries^1–3^. As with the other histologic types of EOC, OCCC frequently presents with various symptoms, including abdominal pain or swelling. In particular, OCCC had been generally associated with ovarian endometriosis, which is characterized by severe dysmenorrhea and chronic pelvic inflammation^4^. Although the majority of OCCC cases are diagnosed in the early-stage and patients with stage IA OCCC have a favorable prognosis, stage IC OCCC with positive peritoneal cytology can lead to poor prognosis due to its high recurrence rate and resistance to conventional platinum-based chemotherapy^5^. Therefore, identification of the clinical indicators that predict long-term outcomes is needed to improve the management of patients with early-stage OCCC.

Malnutrition has been reported to make patients more susceptible to infection, increase the risk of postoperative complications, and promote tumor recurrence through suppression of tumor immunity^6–8^. The prognostic nutritional index (PNI), which is calculated using serum albumin level and lymphocyte count as indicators of nutritional status, has been reported to be correlated with survival and perioperative complications in various types of cancer^8–11^. Even in gynecologic malignancies, low PNI was recently reported to be associated with poor prognosis in HGSOC and cervical cancer^12,13^. Although several reports have shown a correlation between malnutrition and poor prognosis in advanced-stage cancer, the relationship between malnutrition and survival in early-stage cancer has not been sufficiently evaluated. To the best of our knowledge, there has been no report on the correlation of preoperative nutritional status with OCCC prognosis. The aim of this study was to validate the significance of the PNI on the prognosis of patients with early-stage OCCC.

## Results

A total of 82 patients were included in this analysis. The median follow-up period was 63.8 months (range, 2.1–149.6 months). Eight patients died due to disease progression, and 17 patients experienced recurrence.

**Figure 1:**
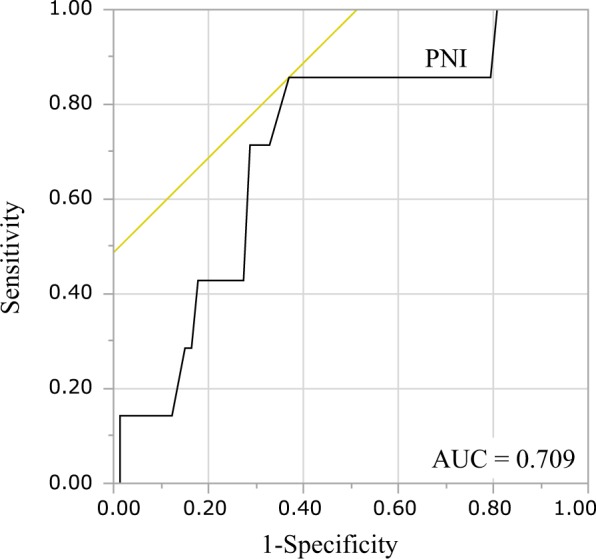
Receiver operating characteristic curve analysis of preoperative PNI. AUC, area under curve; PNI, prognostic nutritional index.

To verify the correlation between nutritional status and OS, and ROC curve for survival was generated (Fig. 1). The AUC for preoperative PNI was 0.709, and the optimal cutoff value for predicting five-year survival was 46.5. The AUC values of albumin and lymphocyte count were 0.647 and 0.678, respectively. The AUC for postoperative long-term prognosis was greater for PNI than for its individual constituents. The determined optimal cutoff value for preoperative PNI had 85.7% sensitivity and 63.1% specificity. Next, the patients were stratified into two groups, based on the optimal cutoff value: the PNI-low (n = 35) and the PNI-high (n = 47) groups.

The clinicopathologic characteristics of all patients stratified into two groups are shown in Table 1. Although age at diagnosis, preoperative PNI, FIGO stage, surgical completeness, status of ascites cytology, TWBC count, platelet count, CA125, type and cycles of adjuvant chemotherapy, and frequency of comorbidities including diabetes, hypertension, and dyslipidemia were similar between the two groups, lymphocyte count, hemoglobin, and albumin were significantly lower in the PNI-low group than in the PNI-high group.

**Table 1:** Patients’ perioperative clinical characteristics stratified by PNI. PNI, prognostic nutritional index; TWBC, total white blood cell; Hb, hemoglobin; SD, standard deviation; NA, not applicable; BMI, body mass index.

	All case (n = 82)	PNI-low (n = 35)	PNI-high (n = 47)	*P*-value
**Age, years**				0.631
mean ± SD	53.45 ± 10.18	51.6 ± 10.3	54.8 ± 10.0	
**Preoperative BMI**				0.148
mean ± SD	21.23 ± 3.63	20.43 ± 2.93	21.84 ± 3.99	
**Stage**				0.05
I	74 (90.2)	29 (82.8)	45 (95.7)	
II	8 (9.8)	6 (17.1)	2 (4.3)	
**Surgery**				0.164
Complete staging laparotomy	56 (68.3)	21 (60.0)	35 (74.4)	
Others	26 (31.7)	14 (40.0)	12 (25.5)	
**Acistes cytology**				0.254
Negative, pseudopositive, or NA	45 (54.9)	23 (65.7)	36 (76.6)	
Positive	23 (28.0)	12 (35.3)	11 (23.4)	
**TWBC count**				0.994
mean ± SD	6.54 ± 1.91	6.54 ± 2.16	6.54 ± 1.72	
**Lymphocyte count**				<0.001
mean ± SD	1.61 ± 0.59	1.31 ± 0.40	1.83 ± 0.61	
**Hb**				<0.001
mean ± SD	12.2 ± 1.52	11.5 ± 1.59	12.7 ± 1.27	
**Platelete**				0.669
mean ± SD	281 ± 32.9	283 ± 33.3	280 ± 32.9	
**Albumin**				<0.001
mean ± SD	3.98 ± 0.49	3.56 ± 0.43	4.27 ± 0.27	
**CA125**				0.908
mean ± SD	197 ± 514	205 ± 341	191 ± 615	
**Adjuvant chemotherapy**				0.120
Palitaxel + Carboplatin	65 (79.3)	29 (82.9)	36 (76.6)	
Docetaxel + Carboplatin	3 (3.7)	0 (0)	3 (6.4)	
Irinotecan + Cisplatin	4 (4.9)	3 (8.6)	1 (2.1)	
No	10 (12.2)	3 (8.6)	7 (14.9)	
**Cycles of adjuvant chemotherapy**				0.380
≤3	35 (42.7)	13 (37.1)	22 (46.8)	
> 3	47 (57.3)	22 (62.9)	25 (53.2)	
**Comorbidities**				
Diabetes	6 (7.3)	2 (5.7)	4 (8.5)	0.627
Hypertension	11 (13.4)	3 (8.6)	8 (17.0)	0.257
Dyslipidemia	10 (12.2)	3 (8.6)	7 (14.9)	0.379

**Figure 2:**
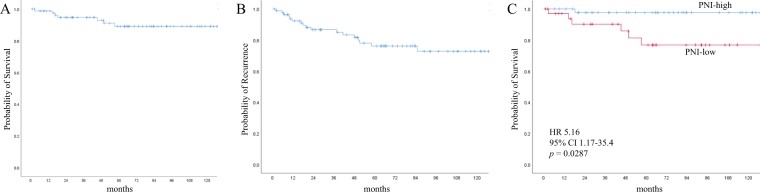
Kaplan–Meier curves of the OS and RFS in all patients, stratified by PNI. The OS (**A**) and RFS (**B**) in all patients and the OS stratified by PNI (**C**). The p-value was calculated by the log-rank test. OS, overall survival; RFS, recurrence-free survival; PNI, prognostic nutritional index; HR, hazard ratio; CI, confidence interval.

To elucidate the prognostic significance of preoperative PNI, we conducted survival analysis. First, we evaluated the prognosis of all patients with early-stage OCCC. On Kaplan–Meier analysis, the 5 year OS and RFS rates were 89.0% and 76.1%, respectively (Fig. 2A,B). Comparing the prognosis of the two groups by the Kaplan–Meier curves, OS was significantly shorter in the PNI-low group than in the PNI-high group (p = 0.028; Fig. 2C). However, the RFS was not significantly different between the PNI-low and PNI-high groups (p = 0.220). The PNI-low and PNI-high groups had 5 year OS rates of 97.6% and 76.8%, respectively, and 5 year RFS rates of 81.9% and 67.1%, respectively.

**Figure 3:**
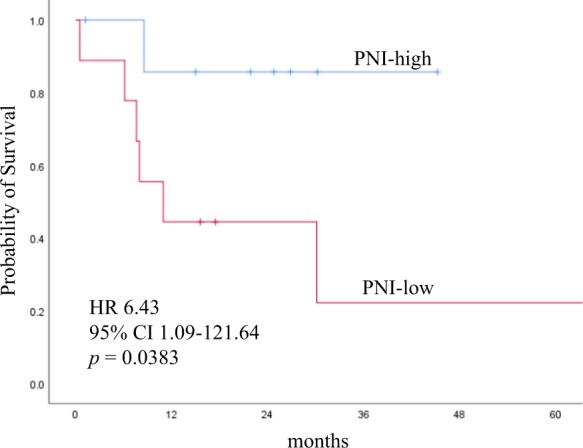
Kaplan–Meier curves of the PRS. The PRS stratified by pretreatment PNI. The p-value was calculated by the log-rank test. PRS, postrecurrence survival; PNI, prognostic nutritional index; CI, confidence interval.

To elucidate the reason for the association between lower preoperative PNI and shorter OS, Kaplan–Meier analysis of the PRS was performed on the 17 recurrent cases. Although there was no significant difference in the PNI at recurrence between the PNI-low and PNI-high groups (p = 0.1043), there was a significant difference in the PRS between the PNI-low and PNI-high groups (p = 0.0383; Fig. 3). Six of 9 (66.7%) patients in the PNI-low group died due to disease progression, whereas only 1 of 8 (11.1%) patients in the PNI-high group died.

Finally, to evaluate the potential prognostic impact of various factors on survival, univariate and multivariate analyses of the clinicopathologic parameters were performed (Table 2). Univariate analysis revealed that PNI was the only predictor of OS. On multivariate analysis, PNI was confirmed as an independent prognostic factor for OS (hazard ratio, 0.102; 95% confidence interval, 0.008–0.602; p < 0.05).

**Table 2:** Univariate and multivariate analyses of the factors for overall survival. PNI, prognostic nutritional index; BMI, body mass idex; SD, standard deviation; NA, not applicable; BMI, body mass index.

	Univariate analysis		Multivariate analysis	
	HR (95% CI)	*P*-value	HR (95% CI)	*P*-value
**Age**		0.596		0.1541
≤55	Reference		Reference	
>55	0.679 (0.137–2.825)		0.306 (0.053–1.577)	
**Preoperative BMI**		0.232		0.249
≤22	Reference		Reference	
>22	0.320 (0.016–1.879)		0.297 (0.014–2.112)	
**Surgery**		0.239		0.091
Complete staging laparotomy	Reference		Reference	
Others	0.326 (0.017–1.884)		0.205 (0.010–1.247)	
**Ascites cytology**		0.471		0.605
Negative, pseudopositive, or NA	Reference		Reference	
Positive	1.716 (0.352–7.001)		1.509 (0.284–6.979)	
**CA125**		0.675		0.941
≤197 U/mL	Reference		Reference	
>197 U/mL	1.425 (0.208–6.206)		1.069 (0.138–5.449)	
**Courses of chemotherapy**		0.124		0.076
≤3	Reference		Reference	
>3	4.090 (0.718–76.816)		5.862 (0.843–117.674)	
**PNI**		0.029		0.010
≤46.5	Reference		Reference	
>46.5	0.193 (0.028–0.848)		0.102 (0.008–0.602)	

## Discussion

This 10 year retrospective study in a single institution revealed that low preoperative PNI ( ≤ 46.5) was an independent poor prognostic factor that was related to short OS and PRS in patients with early-stage OCCC.

The correlation of the indicators reflecting nutritional status with survival in patients with various types of cancer has received much attention in recent years^14–16^. Cancer-related malnutrition is frequently caused by activation of systemic inflammation due to cancer progression, resulting in impaired immunity and reduced survival^8,11,17^. Furthermore, patients with advanced ovarian cancer frequently experience malnutrition due to peritoneal dissemination associated with bowel obstruction^16^. Although the nutritional indicators, including body mass index, C-reactive protein/albumin ratio, lymphocyte/monocyte ratio, modified Glasgow prognostic score, and PNI had been useful predictors of prognosis, PNI has been reported to be superior to the other indicators in ovarian cancer^18^. Among the various indicators, PNI may better reflect both host nutritional and immunologic status and had been validated as an indicator to predict short- and long-term prognosis^14^.

Previous reports have shown a correlation between poor prognosis and low PNI in advanced ovarian cancer, but the significance of PNI in early-stage ovarian cancer has not been evaluated^15^. To interpret the correlation of PNI with prognosis, the fact that the pathogenesis of OCCC frequently originates from endometriosis, which is a chronic inflammatory status, should be considered^19,20^. Prolonged uncontrollable endometriosis can lead to chronic pelvic pain and dysfunctional bowel movements, resulting in poor oral intake and malnutrition. As reported for other cancer types and advanced ovarian cancer, decreased PNI in patients with early-stage OCCC was significantly related with short OS, and this may be attributable to the long-term chronic inflammation from endometriosis.

In this study, we have shown that PNI significantly correlated with OS but not with RFS. This reflected that low preoperative PNI was associated with short PRS. This result suggested that the use of PNI in cases of recurrences would more likely reflect the sensitivity to treatment rather than predict the time to recurrence. Previously, Miao *et al*. reported that PNI was useful for predicting platinum resistance in ovarian cancer and was an independent prognostic factor for OS and progression-free survival^21^. Moreover, Zhang *et al*. reported that decreased PNI correlated with platinum resistance in stage III ovarian cancer^15^. Furthermore, Yoshida *et al*. reported that neutrophil-to-lymphocyte ratio (NLR) at recurrence increased to the same level as preoperative NLR in case of recurrence of OCCC^7^. NLR, as well as PNI, is known as a marker that reflects systemic inflammatory status. Preoperative NLR and PNI may predict tumor inflammation at recurrence. Taken together, our results were consistent with the results of these previous reports, with regard to the correlation between PNI and chemoresistance, and suggested that PNI was a powerful predictor of chemosensitivity in recurrent OCCC.

There had been several reports on the cutoff value of PNI in EOC. Komura *et al*. analyzed 308 patients with EOC and found that a PNI of 44.7 was the optimal cutoff in the early stage and a PNI of 42.9 was the optimal cutoff that had the maximum AUC in advanced EOC^22^. On the other hand, Feng *et al*. reported that a preoperative PNI of <45.45 was associated with advanced stage and platinum resistance in EOC patients^12^. Therefore, the cutoff value of PNI remains controversial. Because the optimal cutoff value could be altered by age and the type, histology, and stage of cancer, this study focused on patients with early-stage OCCC, for which the long-term outcome is difficult to predict upon diagnosis. We found that a PNI of 46.5 demonstrated well-balanced predictive values for OS and PRS in the setting of early-stage OCCC.

Because PNI is calculated only from the serum albumin and peripheral blood lymphocyte count, its measurement can be easily performed in almost all hospitals, without the need for any additional facilities^23^. In addition, PNI can also indicate the need for a more thorough postoperative follow-up for patients who are at high risk for disease recurrence. There had been several compelling reports that supported the opinion that nutritional improvement by preoperative nutritional support may strengthen immunity and promote sensitivity to adjuvant chemotherapy^24,25^. At present, further research is needed to determine whether preoperative nutritional intervention can help increase preoperative PNI and improve the long-term outcomes of patients with OCCC.

There were several limitations in this study. Only 82 patients from a single institution were evaluated, and the retrospective nature of this study could not control the underlying biases. Validation is needed for a definitive conclusion on the prognostic significance of PNI and its optimal cutoff value for early-stage OCCC. Although the results of this study were inconclusive and not definitive, our data suggested that PNI might be a useful indicator of both survival prediction and improvement of nutritional status in early-stage OCCC patients.

This study supported the association of low preoperative PNI with worse prognosis in patients with early-stage OCCC. In conclusion, our study suggested that PNI was a powerful and independent prognostic factor for the long-term survival of patients with early-stage OCCC.

## Materials and Methods

From January 2005 to December 2017, we retrospectively analyzed the data of 82 patients who were diagnosed with OCCC after surgery at the Nagoya University Hospital. This study was approved by the Institutional Review Board (IRB). For this study, the IRB issued a waiver for written informed consent because data collection was retrospective.

The treatment for each patient was determined by several gynecologic oncologists in our hospital, based on comprehensive group discussions and depending on age, disease status, performance status, etc. The standard primary surgical treatment comprised total hysterectomy, bilateral salpingo-oophorectomy, infracolic omentectomy, and systemic retroperitoneal lymphadenectomy. We defined this as the complete staging laparotomy. When patients have severe complications or wish to preserve fertility in reproductive age, we performed conservative surgery, including at least unilateral salpingo-oophorectomy with peritoneal staging. In this study, all patients had no residual tumor after the primary surgery. In principle, all OCCC patients were recommended to receive three to six sessions of adjuvant chemotherapy with a combination of carboplatin [area under the curve (AUC) 5, day 1] and paclitaxel (175 mg/m2, day 1) every 3 to 4 weeks. Patients with alcohol sensitivity underwent docetaxel-based combination therapy. Some patients received irinotecan-cisplatin combination therapy. Post-treatment follow-up was generally done monthly for the first year and was extended after the second year. Recurrence was determined by physical examination; transvaginal ultrasound; blood test findings, including complete blood count and tumor markers; and computed tomography. The treatment for recurrence was surgery or chemotherapy, depending on the recurrent site, number of diseases, etc.

In this study, the preoperative laboratory data, including hemoglobin, total white blood cell count, lymphocyte count, platelet count, albumin, and CA125, were collected from the clinical records within a month prior to surgery. For calculation of the PNI, the following formula was used:

$$10\times {\rm{serum}}\,{\rm{albumin}}\,({\rm{g}}/{\rm{dL}})+0.005\times {\rm{peripheral}}\,{\rm{blood}}\,{\rm{lymphocyte}}\,{\rm{count}}\,({/{\rm{mm}}}^{3})$$

A total of three parameters, including the overall survival (OS), recurrence-free survival (RFS), and postrecurrence survival (PRS), were analyzed. OS was defined as the time between the first surgery and death by any cause. RFS was defined as the time between initial surgery and tumor progression, relapse, or death by any cause. PRS was defined as the time from tumor progression to death. The Response Evaluation Criteria in Solid Tumor criteria were primarily used to evaluate the effects of treatment.

Statistical analyses were performed with the JMP 14.0 (SAS Institute Inc., Cary, NC, USA). Receiver operating characteristic (ROC) curve analysis of OS was performed to determine the optimal cutoff value, which was based on the point on the curve that was within the minimum distance from the left upper corner of the unit square. The baseline characteristics were compared using the qualitative Chi-square test and the quantitative Mann–Whitney U test. Kaplan–Meier method was used for the analyses of OS, RFS, and PRS. Furthermore, p-values were calculated by the log-rank test. To minimize confounding bias, the Cox proportional hazards model was used to identify the independent factors for multivariate analysis. A p-value of <0.05 represented statistical significance.
